# Caesarean-section delivery and caries risk of 3-year-old Chinese children: a retrospective cohort study

**DOI:** 10.1186/s12903-023-02998-w

**Published:** 2023-06-08

**Authors:** Xin Ge, Xiaolin Lyu, Zhifei Zhou, Yang Mi, Tongqiang He, Buling Wu, Fen Liu

**Affiliations:** 1 Department of Stomatology, Xi’an People’s Hospital (Xi’an Fourth Hospital), Northwest University, Xi’an, Shaanxi China; 2 Shenzhen Stomatology Hospital (Pingshan), Southern Medical University, Shenzhen, Guangdong China; 3 Department of Stomatology, General Hospital of Tibetan Military Command, Lhasa, Tibet China; 4 Department of Obstetrics, Northwest Women’s and Children’s Hospital, Xi’an, Shaanxi China; 5 Department of Pediatric Dentistry, College of Stomatology, Xi’an Jiaotong University, Xi’an, Shaanxi China

**Keywords:** Early childhood caries, Retrospective cohort study, Caesarean-section, Primary dentition, Risk factor, Preschool children

## Abstract

**Background:**

Caesarean-section (C-section) may influence children’s long-term health by affecting bacterial colonization. However, few studies have focused on the association between C-section delivery (CSD) and dental caries, and previous conclusions have been conflicting. This study aimed to explore whether CSD would increase the risk of early childhood caries (ECC) in preschool children in China.

**Methods:**

This study was a retrospective cohort study. Three-year-old children with full primary dentition were included through the medical records system. Children in the nonexposure group were vaginally delivered (VD), while children in the exposure group were delivered through C-section. The outcome was the occurrence of ECC. After agreeing to participate in this study, guardians of included children completed a structured questionnaire on maternal sociodemographic factors, children’s oral hygiene and feeding habits. The chi-square test was used to determine differences in the prevalence and severity of ECC between the CSD and VD groups and to analyse the prevalence of ECC according to sample characteristics. Subsequently, potential risk factors for ECC were preliminarily identified through univariate analysis, and the adjusted odds ratios (ORs) were further calculated through multiple logistic regression analysis after controlling for confounding factors.

**Results:**

The VD group included 2115 participants while CSD group included 2996 participants. The prevalence of ECC was higher in CSD children than in VD children (27.6% vs. 20.9%, *P* < 0.05), and the severity of ECC in CSD children was higher (mean number of decayed, missing, and filled teeth, dmft: 2.1 vs. 1.7, *P* < 0.05). CSD was a risk factor for ECC in 3-year-old children (OR = 1.43, 95% CI = 1.10–2.83). In addition, irregular tooth brushing and always prechewing children’s food were risk factors for ECC (*P* < 0.05). Low maternal educational attainment (high school or below) or socioeconomic status (SES-5) may also increase the prevalence of ECC in preschool children and CSD children (*P* < 0.05).

**Conclusions:**

CSD would increase the risk of ECC in 3-year-old Chinese children. Paediatric dentists should devote more attention to the development of caries in CSD children. Obstetricians should also prevent excessive and unnecessary CSD.

## Background

Dental caries occurring in children under 71 months of age are defined as early childhood caries (ECC) [1]. Because of the characteristics of children in this age group, the prevalence of ECC is high [2–5]. The reported prevalence of ECC in China is worrying. Among children aged 3–5 years, the prevalence was 59.0–74.3% in different regions [6, 7]. ECC not only affects the oral health of preschool children but also greatly impacts their quality of life [8]. At present, it is the most prevalent chronic disease that affects the overall health of preschool children [9]. Another factor that substantially impacts the health of children and may impact their risk of ECC is the mode of delivery. Global caesarean-section (C-section) rates are high and increasing. In 2015, an estimated 21.1% or 29.7 million births occurred through C-Sect. [10]. In China, the overall C-section use increased from 40.9% to 2008 to 45.2% in 2018 [11, 12]. C-section delivery is related to early breastfeeding disorder and delayed breastfeeding [13]. Children delivered through C-section are also more likely to suffer from asthma [14], childhood obesity [15] and cardiovascular diseases [16]. In addition, due to changes in the immune system, CSD children also have different inflammatory response characteristics than vaginally delivered (VD) children [17].

CSD can change the intestinal and systemic microflora of new-borns [17]. The diversity of microflora is closely related to general and oral health [18]. VD children exhibit higher rates of probiotics in the oral cavity [19]. Therefore, some researchers have suggested that the prevalence of caries in CSD children may be different from that in VD children [20]. The discovery that *Streptococcus mutans* colonized the oral cavity of CSD children at an earlier age indirectly supports this hypothesis [21]. In a case–control study, the authors also found that more patients with ECC had been delivered through C-Sect. [22]. Thus, CSD is considered as a possible risk factor for ECC.

However, few studies have focused on the prevalence of caries in CSD children, and their conclusions are inconsistent [23, 24]. ECC is affected by many factors, including feeding patterns, oral bacterial colonization or socioeconomic status and lacks targeted preventive measures at present [25]. As mentioned above, in recent years, the elective C-section rate without medical indications has increased; thus, it is of great clinical importance to determine whether this choice impacts children’s long-term oral health. The purpose of this study was to explore the relationship between delivery modes and the prevalence of dental caries in preschool children through a retrospective cohort study and to preliminarily explore other potential risk factors for ECC. Our hypothesis is that the prevalence of ECC in 3-year-old children with full primary dentition would be higher in CSD children than in VD children. The research results are expected to provide theoretical references for paediatric dentists to prevent ECC in CSD populations and to provide data for obstetricians when suggesting a delivery mode.

## Methods

### Study design

This study was a retrospective cohort study. Data were obtained from the Department of Obstetrics (maternal age when pregnant, maternal height and weight in early pregnancy, gestational weeks, delivery mode, infant Apgar score after birth, infant birth weight, complications during pregnancy) and the Department of Stomatology (children’s basic data, dental eruption, and the presence of caries) at Northwest Women’s and Children’s Hospital in Xi’an, Shaanxi Province, China. Through statistical analysis, the influence of CSD and other potential factors on the prevalence of ECC was determined. All guardians of included children were informed of the data use and research purposes by phone or email; if they consented to participate, structured questionnaires were issued to them. Finally, eligible children were included in this study after applying the inclusion criteria (described below). This study was reviewed and approved by the Ethics Committee of Northwest Women’s and Children’s Hospital (IRB-REV-2021-26).

In this study, the exposure factor was delivery by C-section. Children were divided into a nonexposure (VD) group and an exposure (CSD) group. The outcome of this study was the diagnosis of ECC.

### Sample size calculation

A pilot cross-sectional study was carried out in the Department of Stomatology at Northwest Women’s and Children’s Hospital in 2021. Two hundred children aged 3 years old with full primary dentition were randomly selected. Written informed consents were obtained from their parents. In this sample, the prevalence rates of ECC were 28.0% in VD children and 32.0% in CSD children. The sample size for the present study was calculated with 80% power (β = 0.2) and a 5% level of significance (α = 0.05) when detecting difference between groups based on this preliminary data. By using PASS 2021 software (version 21.0.3, NCSS Statistical software, East Kaysville, Utah, USA), the calculation indicated that at least 2057 children should be included in each group.

### Participant recruitment

In this study, all 3-year-old children born from January to December 2019 registered in the medical records system of Northwest Women’s and Children’s Hospital were included. Children who had incomplete eruption of primary dentition or lacked complete oral examination information were excluded. If mothers lacked complete pregnancy examination and delivery data, refused to participate or returned incomplete questionnaires, their children were also excluded. Finally, children were included in the nonexposure (VD) group and the exposure (CSD) group.

### Dental examinations and diagnosis of ECC

Paediatric dentists (GX, XL and FL) who had received standardized training performed dental examinations of the preschool children in dental clinics. Caries was defined as the presence of visible enamel white spots or cavities in the teeth. If necessary, X-ray images were taken to further diagnose the interproximal caries [26]. In children aged 71 months or younger, the presence of caries in one or more teeth (decay, d), missing teeth due to caries (m), or teeth with fillings due to caries (f) resulted in a diagnosis of ECC [27]. Inter-examiner reliability was assessed in a random group of 20 children. The intraclass correlation coefficient was 0.97 (*P* < 0.001). Regarding intra-examiner agreement, 10 same patients were examined twice by one examiner at an interval of 1 week. The results showed a high intra-examiner agreement for each dentist (the intraclass correlation coefficients were 0.94, 0.98 and 0.98, respectively, for GX, XL and FL; *P* < 0.001).

### Collection of pregnancy and delivery information

In the medical records system of Northwest Women’s and Children’s Hospital, the maternal pregnancy and delivery information of each included mother-child pair was extracted, including maternal age when pregnant, maternal height and weight in early pregnancy, occurrence of pregnancy complications (hypertension or gestational diabetes mellitus), gestational weeks, delivery mode, infant Apgar score after birth and infant birth weight. Maternal body mass index (BMI) was calculated by dividing height (m) by the square of weight (kg). In this study, BMI was categorized as ≥ 25 kg/m^2^ or < 25 kg/m^2^. The exposure factor in this study was CSD, which was defined as the decision to undergo delivery by C-section determined before the start of uterine contractions and included elective C-section and emergency C-section. This study did not include women who elected to undergo delivery by C-section after failure of vaginal delivery.

### Structured questionnaires

The structured questionnaire was distributed or mailed to the mothers of included children and then collected. In the structured questionnaire, questions on children’s characteristics included the main feeding patterns before 6 months of age (breastfeeding, mainly breastfeeding, mainly bottle feeding, or bottle feeding) and whether their teeth were brushed regularly (regularly, frequently, or occasionally). Questions related to maternal characteristics included whether smoking during pregnancy (yes/no) and the frequency of prechewing food before giving it to children (prechewing feeding habits: always, occasionally, or never). In addition, to calculate socioeconomic status (SES) [28], maternal educational attainment, occupation and annual income were collected. Based on the scores, SES was divided into five levels (1: Upper Class; 2: Upper Middle Class; 3: Middle Class; 4: Lower Middle Class; 5: Lower Class). In this study, educational attainment was divided into graduate or above, undergraduate only, and senior high school or below to simplify categorization. Cronbach’s α coefficient was calculated for this questionnaire, and the result of 0.92 indicated a favorable reliability for this questionnaire. In this study, we sent out a total of 5051 questionnaires and received 4411 qualified ones (87.3%), showing a good acceptability for this questionnaire.

### Statistical analysis

SPSS software (version 22.0, IBM, Armonk, NY, USA) was used to compile and analyse the data. The association between CSD and the prevalence of ECC was estimated by the chi-square test and logistic regression analysis, and the odds ratio (OR) and 95% confidence interval (CI) of potential risk factors were calculated. *P* < 0.05 (two tailed) indicated that a difference was statistically significant.

In this retrospective cohort study, the main exposure factor was CSD, and the association between CSD and the prevalence of ECC was determined by univariate and multivariate analyses. The outcome was the occurrence of ECC; children were divided into those with ECC and those without ECC. When multiple logistic regression was used to determine the association between CSD and ECC, other factors that might affect the occurrence of ECC and factors that differed between groups were controlled as confounding factors. To prevent the exclusion of possible confounding factors, *P* < 0.1 were considered statistically significant when screening factors that might affect the development of ECC [29].

## Results

### Participants of the groups

**Fig. 1:**
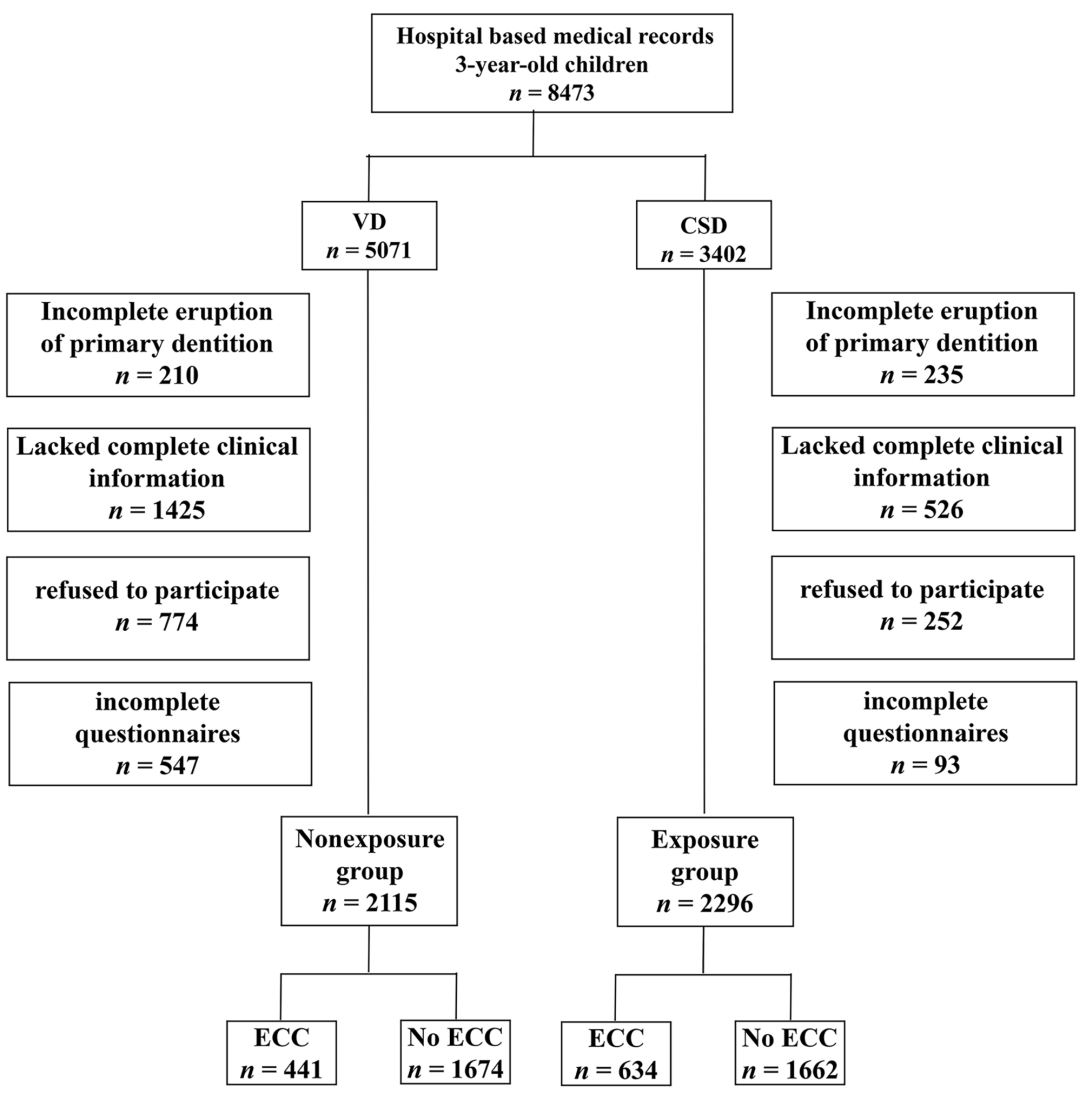
Sample inclusion in this retrospective cohort study Three-year-old children were identified from the medical records system. After excluding children with incomplete eruption of primary teeth or lacked complete dental clinical information, and parents refused to participate or filled incomplete questionnaires, the nonexposure (VD) group included 2115 children (with 441 ECC patients), while the exposure group (CSD) included 2296 children (with 634 ECC patients). CSD: caesarean-section delivery; ECC: early childhood caries; VD: vaginal delivery

At the beginning of this study, 8473 3-year-old children were eligible from the medical records system of Northwest Women’s and Children’s Hospital. Of these children, 5071 were VD children, and 3402 were CSD children. Of the VD children, 210 had incomplete eruption of primary dentition, 359 lacked complete oral examination information; 1066 mothers lacked complete pregnancy examination and delivery data, 774 mothers refused to participate, and 547 mothers returned incomplete questionnaires. Of the CSD children, 235 had incomplete eruption of primary dentition, 227 lacked complete oral examination information, 299 mothers lacked complete pregnancy examination and delivery data, 252 mothers refused to participate, and 93 mothers returned incomplete questionnaires. Finally, 2115 children were included in the nonexposure (VD) group, and 2296 children were included in the exposure (CSD) group (Fig. 1).

### Child and maternal characteristics of the groups

A total of 1863 children (88.1%) in the VD group were born after 37 weeks of gestation, while 1843 children (80.3%) in the CSD group were born after 37 weeks of gestation. The proportion of children born before 37 weeks of gestation was significantly higher in the CSD group than in the VD group (19.7% vs. 11.9%; *P* < 0.05; Table 1). Apgar scores were collected 5 min after birth (5-min Apgar scores). The proportion of children with low Apgar scores was significantly higher in the CSD group than in the VD group (20.0% vs. 13.0%; *P* < 0.05; Table 1). In addition, the probability of low birth weight was significantly higher in the CSD group (8.2%) than in the VD group (6.2%; *P* < 0.05; Table 1). There were no significant differences between the two groups in terms of sex, feeding patterns before 6 months of age and habits of brushing teeth (Table 1, *P* > 0.05).

**Table 1:** Characteristics of children with vaginal or caesarean-section delivery as well as maternal characteristics

	Non-exposure group VD (%)	Exposure group CSD (%)	χ^2^	*P* value
**Total**	2115	2296		
**Child characteristics**				
Sex				
Male	1128 (53.3)	1225 (53.4)		
Female	987 (46.7)	1071 (46.6)	0.0002	0.99
Gestational weeks				
≥ 37	1863 (88.1)	1843 (80.3)		
< 37	252 (11.9)	453 (19.7)	49.49	< 0.001
5-min Apgar scores				
Normal	1840 (87.0)	1837 (80.0)		
Low	275 (13.0)	459 (20.0)	38.77	< 0.001
Birth weight, g				
> 4000	508 (24.0)	536 (23.3)		
2500–4000	1476 (69.8)	1571 (68.4)		
< 2500	131 (6.2)	189 (8.3)	6.81	0.03
Feeding patterns before 6 months of age				
Breastfeeding	302 (14.3)	318 (13.9)		
Mainly breastfeeding	902 (42.7)	1060 (46.2)		
Mainly bottle feeding	790 (37.4)	777 (33.8)		
Bottle feeding	121 (5.6)	141 (6.1)	7.36	0.06
Tooth brushing				
Regular	662 (31.3)	722 (31.5)		
Frequent	1082 (51.2)	1172 (51.1)		
Occasional	371 (17.5)	402 (17.4)	0.01	0.99
**Maternal characteristics**				
Age when pregnant, years				
> 34	465 (22.0)	735 (32.0)		
25–34	1417 (64.0)	1424 (62.0)		
< 25	233 (14.0)	137 (6.0)	78.38	< 0.001
Body mass index in early pregnancy, kg/m^2^				
≥ 25	465 (22.0)	593 (25.8)		
< 25	1650 (78.0)	1703 (74.2)	8.91	0.003
Hypertension during pregnancy				
Yes	99 (4.7)	119 (5.2)		
No	2016 (95.3)	2177 (94.8)	0.59	0.44
Gestational diabetes mellitus				
Yes	101 (4.8)	148 (6.5)		
No	2014 (95.2)	2148 (93.5)	5.77	0.02
Smoking during pregnancy				
Yes	148 (7.0)	161 (7.0)		
No	1967 (93.0)	2135 (93.0)	0.0004	0.99
Educational attainment				
Graduate or above	359 (17.0)	430 (18.7)		
Undergraduate	873 (41.3)	1057 (46.0)		
High school or below	883 (41.7)	809 (35.3)	19.77	< 0.001
Social economic status (SES)				
SES-1	328 (15.5)	389 (16.9)		
SES-2	871 (41.2)	956 (41.6)		
SES-3	557 (26.3)	543 (23.7)		
SES-4	140 (6.6)	162 (7.1)		
SES-5	219 (10.4)	246 (10.7)	5.07	0.28
Prechewing feeding habit				
Always	207 (9.8)	229 (10.0)		
Occasionally	622 (29.4)	685 (29.8)		
Never	1286 (60.8)	1382 (60.2)	0.17	0.92

In terms of maternal characteristics, 735 mothers (32.0%) in the CSD group were older than 34 years when pregnant, a higher proportion than that in the VD group (465 mothers, 22.0%). Additionally, the proportion of mothers with a BMI ≥ 25 kg/m^2^ in early pregnancy was significantly higher in the CSD group than in the VD group (25.8% vs. 22.0%; *P* < 0.05; Table 1). In terms of pregnancy complications, the prevalence of gestational diabetes mellitus was significantly higher in the CSD group than in the VD group (Table 1, *P* < 0.05). Maternal educational attainment was greater in the CSD group than in the VD group; in the CSD group, 64.8% of mothers had an undergraduate degree or above, and the proportion of people with a high school education or below was low (35.2% vs. 41.8% in the CSD group vs. VD group, respectively). In addition to the above factors, there were no significant group differences in the prevalence of hypertension or smoking during pregnancy, maternal SES or prechewing feeding habits (Table 1, *P* > 0.05).

### Group differences in the prevalence of ECC

The prevalence of ECC in CSD children was 27.6% and that in VD children was 20.9% (Fig. 1). The prevalence of ECC in children in the CSD group was higher than that in children in the VD group (Table 2, *P* < 0.05). The crude OR of the influence of CSD on the prevalence of ECC was 1.45 (95% CI = 1.26–1.66). The mean dmft was higher in CSD children than in the VD group (0.6 ± 2.0 vs. 0.5 ± 1.2, *P* < 0.05). Further analysis of the mean dmft among ECC children showed that the mean dmft was higher in CSD children than in VD children (Table 2, 2.1 ± 2.1 vs. 1.7 ± 1.7, *P* < 0.05).

**Table 2:** Prevalence of ECC in children with vaginal delivery (VD) and caesarean-section delivery (CSD)

	VD group	CSD group	χ^2^/*t*	*P* value
Total	2115	2296		
ECC (%)	441 (20.9)	634 (27.6)	26.95^*^	< 0.001**
dmft (overall participants)	0.5 ± 1.2	0.6 ± 2.0	2.44^***^	0.02
dmft (ECC children)	1.7 ± 1.7	2.1 ± 2.1	3.92^***^	< 0.001

* Chi-square test; ** crude odds ratio (OR): 1.45, 95% confidence interval: 1.26 to 1.66; *** Student’s t test. ECC: early childhood caries; dmft: numbers of decayed, missing and filled primary teeth

Results further showed the prevalence of ECC according to sample characteristics (Table 3). The prevalence of ECC was higher in CSD children than in VD children of both sexes or different birth weights (*P* < 0.05). In addition, the prevalence of ECC was the lowest in children with the highest SES (SES-1), at 11.9% in the VD group and 16.2% in the CSD group; these groups did not significantly differ. The prevalence of ECC was the highest in CSD children at SES-4, reaching 35.2%.

**Table 3:** Prevalence of early childhood caries according to the characteristics of children and mothers

Characteristics	VD group (441/2115)	CSD group (634/2296)	χ^2^	*P* value
Child characteristics				
Sex				
Male	234 (20.7%)	344 (28.1%)	17.06	< 0.001
Female	207 (21.0%)	290 (27.1%)	10.45	0.001
Gestational weeks				
≥ 37	381 (20.5%)	520 (33.8%)	30.35	< 0.001
< 37	60 (23.8%)	114 (25.2%)	0.16	0.69
5-min Apgar scores				
Normal	381 (20.7%)	513 (27.9%)	26.04	< 0.001
Low	60 (21.8%)	121 (26.4%)	2.39	0.12
Birth weight, g				
> 4000	102 (20.1%)	167 (31.2%)	16.73	< 0.001
2500–4000	315 (21.3%)	410 (26.1%)	9.50	0.002
< 2500	24 (18.3%)	57 (30.2%)	5.74	0.02
Feeding patterns before 6 months of age				
Breastfeeding	56 (18.5%)	82 (25.8%)	4.70	0.03
Mainly breastfeeding	187 (20.7%)	284 (26.8%)	9.81	0.002
Mainly bottle feeding	171 (21.7%)	229 (29.5%)	12.62	< 0.001
Bottle feeding	27 (22.3%)	39 (27.7%)	0.99	0.32
Tooth brushing				
Regular	125 (18.9%)	154 (21.3%)	1.29	0.26
Frequent	228 (21.1%)	368 (31.4%)	30.85	< 0.001
Occasional	88 (23.7%)	112 (27.9%)	1.73	0.19
**Maternal characteristics**				
Age when pregnant, years				
> 34	92 (19.8%)	211 (28.7%)	12.01	< 0.001
25–34	297 (21.0%)	388 (27.3%)	15.34	< 0.001
< 25	52 (22.3%)	35 (25.6%)	0.50	0.48
Body mass index in early pregnancy, kg/m^2^				
≥ 25	127 (27.3%)	182 (30.7%)	1.44	0.23
< 25	314 (19.0%)	452 (26.5%)	26.82	< 0.001
Hypertension during pregnancy				
Yes	20 (20.2%)	24 (20.2%)	0.00	0.99
No	421 (20.9%)	610 (28.0%)	28.76	< 0.001
Gestational diabetes mellitus				
Yes	20 (19.8%)	37 (25.0%)	1.80	0.18
No	421 (20.9%)	597 (27.8%)	26.70	< 0.001
Smoking during pregnancy				
Yes	42 (28.4%)	39 (24.2%)	0.69	0.41
No	399 (20.3%)	595 (27.9%)	32.07	< 0.001
Educational attainment				
Graduate or above	60 (16.7%)	103 (24.0%)	6.26	0.01
Undergraduate	150 (17.2%)	293 (27.7%)	30.02	< 0.001
High school or below	231 (26.2%)	238 (29.4%)	2.24	0.14
Social economic status (SES)				
SES-1	39 (11.9%)	63 (16.2%)	2.70	0.10
SES-2	213 (24.5%)	280 (29.3%)	5.41	0.02
SES-3	114 (20.5%)	172 (31.7%)	17.96	< 0.001
SES-4	31 (22.1%)	57 (35.2%)	6.19	0.01
SES-5	44 (20.1%)	62 (25.2%)	1.72	0.19
Prechewing feeding habit				
Always	51 (24.6%)	78 (34.1%)	4.63	0.03
Occasionally	152 (24.4%)	182 (26.6%)	0.78	0.38
Never	238 (18.5%)	374 (27.1%)	27.58	< 0.001

VD: vaginal delivery; CSD: caesarean-section delivery

### Risk factors for ECC

After establishing group differences in the prevalence of ECC, we next aimed to identify risk factors for ECC. First, a univariate analysis was performed to compare characteristics of children with and without ECC (Table 4). To include all potential confounding factors, *P* < 0.1 was set as the threshold for statistical significance. The results suggested that habits of brushing teeth, maternal BMI in early pregnancy, maternal educational attainment, maternal SES, and prechewing feeding habits were potential risk factors for ECC aside from CSD.

**Table 4:** Univariate analysis of potential risk factors affecting the prevalence of early childhood caries (ECC)

Characteristics	Without ECC	ECC	χ2	*P* value
**Child characteristics**				
Sex				
Male	1775	578		
Female	1561	497	0.10	0.75
Gestational weeks				
≥ 37	2805	901		
< 37	531	174	0.04	0.83
5-min Apgar scores				
Normal	2783	894		
Low	553	181	0.04	0.84
Birth weight, g				
> 4000	775	269		
2500–4000	2322	725		
< 2500	239	81	1.81	0.41
Feeding patterns before 6 months of age				
Breastfeeding	482	138		
Mainly breastfeeding	1491	471		
Mainly bottle feeding	1167	400		
Bottle feeding	196	66	2.87	0.41
Tooth brushing				
Regular	1105	279		
Frequent	1658	596		
Occasional	573	200	19.51	< 0.001
**Maternal characteristics**				
Age when pregnant, years				
> 34	897	303		
25–34	2156	685		
< 25	283	87	0.75	0.69
Smoking during pregnancy				
Yes	228	81		
No	3108	994	0.61	0.43
Body mass index in early pregnancy, kg/m^2^				
≥ 25	749	309		
< 25	2587	766	17.65	< 0.001
Educational attainment				
Graduate or above	626	163		
Undergraduate	1487	443		
High school or below	1223	469	18.29	< 0.001
Social economic status (SES)				
SES-1	615	102		
SES-2	1334	493		
SES-3	814	286		
SES-4	214	88		
SES-5	359	106	52.74	< 0.001
Hypertension during pregnancy				
Yes	174	44		
No	3162	1031	2.18	0.14
Gestational diabetes mellitus				
Yes	192	57		
No	3144	1018	0.31	0.58
Prechewing feeding habit				
Always	307	129		
Occasionally	973	334		
Never	2056	612	10.40	0.01

Significant variables identified in the univariate analysis were included in the multiple logistic regression model to identify risk factors for ECC. When analysing the impact of one factor on the risk of ECC, the other factors and the five factors that were different between groups at baseline (gestational weeks, 5-min Apgar score, birth weight, maternal age when pregnant, and presence of gestational diabetes mellitus) were controlled as confounding factors (Table 5). The results suggested that CSD was a risk factor for ECC. Compared with VD children, CSD children had a 1.43-fold higher risk of ECC (95% CI = 1.10–2.83). In addition, the OR of ECC in children with irregular brushing of teeth was 1.87 (95% CI = 1.54–2.60) compared to children with regular brushing of teeth. Maternal educational attainment in high school and below and always prechewing food were also risk factors for ECC, with ORs of 1.57 and 1.68, respectively (Table 5, *P* < 0.05). Thus, risk factors for ECC in the overall sample included CSD, irregular brushing of teeth, low maternal educational attainment, and always prechewing food.

**Table 5:** Adjusted ORs of factors affecting the prevalence of ECC by multiple logistic regression analysis

Characteristics	B	SE	*P* value	Adjusted OR	95% Confidence interval	
					Lower limit	Upper limit
Vaginal delivery				1.00 (reference)		
Caesarean-section delivery	0.38	0.03	0.02	1.43	1.10	2.83
Regular brushing of teeth				1.00 (reference)		
Frequent	0.37	0.27	0.57	1.42	0.86	1.75
Occasional	0.54	0.14	0.01	1.87	1.54	2.60
Educational attainment: graduate or above				1.00 (reference)		
Undergraduate	0.27	0.14	0.50	1.23	0.98	1.64
High school and below	0.46	0.09	0.001	1.57	1.25	2.19
Social economic status (SES)-1				1.00 (reference)		
SES-2	0.34	0.30	0.94	1.32	0.64	1.68
SES-3	0.22	0.14	0.08	1.01	0.76	1.33
SES-4	0.42	0.25	0.14	1.55	0.76	2.28
SES-5	0.35	0.22	0.51	1.40	0.91	1.75
No prechewing feeding habit				1.00 (reference)		
Occasionally	0.35	0.30	0.10	1.39	0.75	1.92
Always	0.50	0.06	0.05	1.68	1.43	2.41

OR: odds ratio; ECC: early childhood caries

### Risk factors for ECC among CSD children

Similarly, a univariate analysis was used to identify characteristics that differed between children with and without ECC in the CSD group (Table 6). To include all potential confounding factors, *P* < 0.1 was also used as the threshold for statistical significance. The results suggested that the risk factors for ECC in CSD children may be high birth weight, irregular brushing of teeth, maternal BMI in early pregnancy, SES, hypertension during pregnancy and prechewing feeding habits.

**Table 6:** Univariate analysis of potential risk factors affecting the prevalence of ECC in CSD children

Characteristics	Without ECC	ECC	χ^2^	*P* value
Child characteristics				
Sex				
Male	881	344		
Female	781	290	0.29	0.54
Gestational weeks				
≥ 37	1323	520		
< 37	339	114	1.69	1.30
5-min Apgar scores	338	121	0.45	0.50
Normal	2998	954		
Low	338	121	0.45	0.50
Birth weight, g				
> 4000	369	167		
2500–4000	1161	410		
< 2500	132	57	5.78	0.06
Feeding patterns before 6 months of age				
Breastfeeding	236	82		
Mainly breastfeeding	776	284		
Mainly bottle feeding	548	229		
Bottle feeding	102	39	2.23	0.53
Tooth brushing				
Regular	568	154		
Frequent	804	368		
Occasional	290	112	22.68	< 0.001
**Maternal characteristics**				
Age when pregnant, years				
> 34	524	211		
25–34	1036	388		
< 25	102	35	0.83	0.66
Body mass index in early pregnancy, kg/m^2^				
≥ 25	411	182		
< 25	1251	452	3.79	0.05
Hypertension during pregnancy				
Yes	95	24		
No	3241	1051	3.48	0.06
Gestational diabetes mellitus				
Yes	111	37		
No	3225	1038	0.54	0.46
Smoking during pregnancy				
Yes	122	39		
No	3214	1036	1.00	0.32
Educational attainment				
Graduate or above	327	103		
Undergraduate	764	293		
High school or below	571	238	4.21	0.12
Social economic status (SES)				
SES-1	326	63		
SES-2	676	280		
SES-3	371	172		
SES-4	105	57		
SES-5	184	62	36.56	< 0.001
Prechewing feeding habit				
Always	151	78		
Occasionally	503	182		
Never	1008	374	5.35	0.07

ECC: early childhood caries; CSD: caesarean-section delivery

These factors were included in the multiple logistic regression analysis. When analysing the impact of one of these factors on the occurrence of ECC, other factors were controlled as confounding factors (Table 7). The results suggested that similar to the findings in the overall sample, irregular brushing of teeth and prechewing feeding habits were risk factors for ECC in CSD children. Compared with CSD children with regular brushing of teeth, those with irregular brushing of teeth were 1.48 times more likely to suffer from ECC (95% CI = 1.06–2.23). In CSD children, mothers always feeding their children prechewed food had an OR of 1.51 (95% CI = 1.25–2.02). Additionally, in the CSD children, if maternal SES was SES-5, the risk of ECC was 1.56 times higher than if maternal SES was SES-1 (Table 7, *P* < 0.05).

**Table 7:** Adjusted ORs of factors affecting the prevalence of ECC by multiple logistic regression analysis in CSD children

Characteristics	B	SE	*P* value	Adjusted OR	95% Confidence interval	
					Lower limit	Upper limit
Birth weight: 2500–4000 g				1.00 (reference)		
> 4000	0.43	0.25	0.10	1.35	0.73	2.12
< 2500	0.61	0.36	0.17	1.83	0.61	2.13
Regular brushing of teeth				1.00 (reference)		
Frequent	0.49	0.33	0.39	1.57	0.58	2.11
Occasional	0.46	0.14	0.02	1.48	1.06	2.23
BMI < 25 kg/m^2^ in early pregnancy				1.00 (reference)		
≥ 25	0.43	0.30	0.72	1.34	1.19	1.84
Social economics status (SES)-1				1.00 (reference)		
SES-2	0.33	0.11	0.07	1.20	0.08	1.63
SES-3	0.50	0.37	0.18	1.61	0.82	2.25
SES-4	0.31	0.16	0.07	1.11	0.60	2.04
SES-5	0.48	0.11	0.01	1.56	1.18	1.93
No hypertension during pregnancy				1.00 (reference)		
Hypertension	-0.48	0.41	0.55	0.57	0.09	1.47
No prechewing feeding habit				1.00 (reference)		
Occasionally	0.58	0.57	0.23	1.80	0.69	2.60
Always	0.47	0.14	0.03	1.51	1.25	2.02

OR: odds ratio; ECC: early childhood caries; CSD: caesarean-section delivery; BMI: body mass index

## Discussion

This study was a retrospective cohort study focusing on the possible relationship between different delivery modes and ECC occurrence. Results indicated that the prevalence of ECC was higher in CSD children than in VD children, and the severity of ECC was also higher in CSD children. CSD was an important risk factor for ECC in 3-year-old children with full primary dentition.

In a study on differences in oral bacterial colonization between VD and CSD children, researchers collected saliva samples from children from birth to 4 years of age in a prospective cohort; they found that CSD children exhibited earlier colonization of *Streptococcus mutans* [21] but that the detection rate of health-promoting Streptococcus and Lactobacillus species was higher in VD children than in CSD children [19]. Furthermore, in a study using a human oral microbe identification microarray, researchers found that among 3-month-old infants, the diversity of oral microorganisms was better in VD children than in CSD children; in contrast, *Slackia exigua*, a gram-positive anaerobic bacterium, was detected only in CSD children [18].

Due to the difference in bacterial and microbial composition, researchers have speculated that children with different delivery modes may have different risks of dental caries. Because it may affect the composition of *Streptococcus mutans* and other oral microorganisms, CSD could increase the risk of dental caries in preschool children. The results of the present study support this theory. Even after controlling for other potential confounding factors, CSD may also be a risk factor for ECC in 3-year-old children with full primary dentition (OR = 1.43, *P* < 0.05). This finding is consistent with those of Peretz and other researchers [22]. They found that the occurrence of baby bottle caries was closely related to the delivery mode. However, other researchers have reached different conclusions. In a retrospective cohort study also focusing on 3-year-old children, researchers found no difference in the prevalence of ECC between CSD children and VD children [24]; however, the author noted that CSD may increase the severity of disease in ECC patients. This finding is consistent with that of a retrospective cohort study conducted by another Brazilian researcher on preschool children aged 2–6 years [30]. However, some researchers have suggested that CSD may be a protective factor against ECC [23, 31, 32]. In our analysis, we noticed that some studies lacked an evaluation of the sample size before the study, and there were large differences in the selection of disease-relevant variables. Because many factors affect the development of caries, especially ECC [33], it is difficult to include all these factors within a single study. Research findings can also be influenced by differences in the research methods (e.g., retrospective cohort study or cross-sectional study), research objects (age groups and ethnicity), calculation of necessary sample size, and selection and control of confounding factors (logistic regression or marginal structural model). Thus, prospective studies with large sample sizes that assess underlying mechanisms of microbial colonization are needed.

In this study, we defined CSD as a maternal choice of delivery mode before the start of delivery, rather than after vaginal delivery did not progress. Because during VD, the foetus may enter the birth canal and come into contact with maternal vaginal bacteria due to the rupture of the amniotic sac. We excluded children that may have been exposed in this manner, as the mechanism by which CSD is thought to influence ECC in children is to interfere with initial bacterial colonization.

In addition to bacterial factors, living habits are also an important factor for the occurrence of ECC [33]. In particular, feeding habits have attracted attention from researchers. Although the mechanism remains unclear, previous studies have shown that breastfeeding is an important protective factor against ECC [34]. A possible explanation is that immunoglobulins in breast milk have been found to protect against the development of *Streptococcus mutans*, which leads to primary tooth decay [35]. The breastfeeding rate of CSD children was found to be lower than that of VD children [36]. In addition, the breastfeeding period of women who underwent elective CSD was also shorter than that of women who underwent VD [37]. Therefore, the difference in feeding habits is another possible explanation for the differences in the prevalence of ECC between the two delivery modes. However, some researchers have noted that the protective effect of breastfeeding against ECC is limited to infants under 12 months old. Repeated and prolonged exposure to breastfeeding over 12 months after birth would increase the incidence of caries, as it may lead to a reduction in plaque pH and thus, the decalcification of tooth enamel [38]. Thus, the relationship between breastfeeding and caries is complex, and there are many influencing factors. The specific interaction effects still need to be identified.

In this study, our results did not suggest that feeding patterns affected the prevalence of ECC. However, we found that in both the whole sample (OR = 1.68) and in CSD children (OR = 1.51), always prechewing food before feeding it to children increased the prevalence of ECC. Prechewing children’s food may transmit cariogenic bacteria from the mother’s mouth to the child’s mouth. Previous study has also confirmed that maternal prechewing feeding habits increase the colonization of *Streptococcus mutans* in children’s oral cavity [23]. This result partly supports the findings of our study.

Sociodemographic factors also play important roles in the occurrence of childhood caries [39]. The results of the present study suggest that children of mothers with high school education or below may have an increased risk of ECC (OR = 1.57). In CSD children, the risk of ECC was also increased (OR = 1.56) when maternal SES was the lowest (SES-5). We calculated the SES level using a method that incorporates occupation, educational attainment and annual income [28]. In addition to educational attainment, family income is also one of the possible factors that affect the development of caries [40]. Therefore, the results of this study partly confirmed previous proposals of a relationship between sociodemographic characteristics and ECC. In further studies, larger samples with more rigorous methods are needed to explore this topic.

This study also has some limitations. First, as a retrospective study, it lacks in-depth assessment of the underlying mechanisms, such as the detection of oral microorganisms. Further mechanistic studies are needed to support these findings. In addition, in terms of the selection of variables, we attempted to include all potential risk factors related to caries in this study. However, numerous factors affect the development of caries, including the use of antibiotics, systemic diseases, and dietary habits. Thus, further explorations are also needed on the influencing factors of ECC. Finally, given the retrospective nature of this study, there may have been recall bias in the questionnaire results. However, as mentioned previously, this questionnaire still has favorable intrinsic validity (containing positive indicators), reliability (Cronbach’s α coefficient equals 0.92) and acceptability (87.3% qualified questionnaires received).

## Conclusions

In summary, the results of this retrospective cohort study suggest that CSD would increase the risk of ECC in 3-year-old Chinese children. Paediatric dentists should devote more attention to the development of caries in CSD children. Obstetricians should also prevent excessive and unnecessary CSD.

## Data Availability

The datasets used and/or analysed during the current study are available from the corresponding author on reasonable request.

## Abbreviations

BMI: Body mass index
CI: Confidence interval
CSD: Caesarean-section delivery
dmft: Decayed, missing and filled primary teeth
ECC: Early childhood caries
OR: Odds ratio
SES: Socioeconomic status
VD: Vaginal delivery
